# Supplementary material for: Chemical Nature of Metals and Metal-Based Materials in Inactivation of Viruses
Source: Nanomaterials (Basel). 2022 Jul 8;12(14):2345. doi: 10.3390/nano12142345 (PMC9323642; doi:10.3390/nano12142345)
Supplement: Supplementary file 1 [file nanomaterials-12-02345-s001.zip › nanomaterials-1763407-supplementary.pdf]

Supplementary Materials

Chemical Nature of Metals and Metal-Based Materials in Inactivation of Viruses

Haozhong Tian [1,2], Bin He [1,2,3], Yongguang Yin [1,2,3], Lihong Liu [1], Jianbo Shi [1,2,4], Ligang Hu [1,2,3,4,*] and Guibin Jiang [1,2,3]

1 State Key Laboratory of Environmental Chemistry and Ecotoxicology, Research Center for Eco-Environmental Sciences, Chinese Academy of Sciences, 18 Shuangqing Road, Beijing 100085, China; hztian_st@rcees.ac.cn (H.T.); bhe@rcees.ac.cn (B.H.); ygyin@rcees.ac.cn (Y.Y.); lhliu@rcees.ac.cn (L.L.); jbshi@rcees.ac.cn (J.S.); gbjiang@rcees.ac.cn (G.J.)
2 College of Resources and Environment, University of Chinese Academy of Sciences, Beijing 100049, China
3 School of Environment, Hangzhou Institute for Advanced Study, University of Chinese Academy of Sciences, Hangzhou 310024, China
4 School of Environment and Health, Jianghan University, Wuhan 430056, China
* Correspondence: author: lghu@rcees.ac.cn

Table S1. Selected the chelation equilibrium constants between M^{n+} and EDTA.

Atomic number	Metal ion	lg K_0	Atomic number	Metal ion	lg K_0
19	K^{+}	1.51[a]	30	Zn^{2+}	18.22
11	Na^{+}	2.72	48	Cd^{2+}	18.24
3	Li^{+}	3.71	13	Al^{3+}	19.10
47	Ag^{+}	8.01	28	Ni^{2+}	20.14
29	Cu^{+}	9.15	29	Cu^{2+}	20.40
56	Ba^{2+}	9.43	22	Ti^{3+}	23.82
12	Mg^{2+}	10.43	24	Cr^{3+}	25.77
20	Ca^{2+}	12.39	22	Ti^{4+}	26.63
23	V^{2+}	14.34	25	Mn^{3+}	27.23
25	Mn^{2+}	15.54	26	Fe^{3+}	27.43
26	Fe^{2+}	15.86	23	V^{3+}	28.37
27	Co^{2+}	17.98	27	Co^{3+}	43.78
50	Sn^{2+}	18.17			

[a] All data were measured under standard conditions (273.15 K, 101 kPa).

Table S2. Antiviral rate of various M^{n+}.

Metal ion	lg K_0	Antiviral rate ($mM^{-1}\cdot h^{-1}$)				Mean
		Example A	Example B	Example C	Example D	
K^+	1.51	0				0
Na^+	2.72	0				0
Li^+	3.71	0.003 [1]	0.005 [2]	0.005 [3]	0.080 [4]	0.023
Ag^+	8.01	0.101 [5]	0.750 [5]	2.080 [6]		0.977
Cu^+	9.15	0.314 [7]	1.500 [8]	6.190 [9]		2.668
Ba^{2+}	9.43	0				0
Mg^{2+}	10.43	0 [10]	0.064 [11]			0.032
Ca^{2+}	12.39	0				0
V^{2+}	14.34	0				0
Mn^{2+}	15.54	0				0
Fe^{2+}	15.86	0.090 [12]	0.605 [6]			0.348
Co^{2+}	17.98	0				0
Sn^{2+}	18.17	0				0
Zn^{2+}	18.22	0 [13]	0.135 [14]	0.169 [11]	0.250 [15]	0.138
Cd^{2+}	18.24	0				0
Al^{3+}	19.1	0 [10]	0.343 [6]			0.171
Ni^{2+}	20.14	0 [16]	0.033 [6]			0.016
Cu^{2+}	20.4	0.029 [5]	1.100 [6]	5.083 [17]		2.070
Ti^{3+}	23.82	0				0
Cr^{3+}	25.77	0 [18]	0.114 [18]			0.057
Ti^{4+}	26.63	0				0
Mn^{3+}	27.23	0				0
Fe^{3+}	27.43	0.006 [18]	0.041 [18]			0.023
V^{3+}	28.37	0				0

Table S3. Selected the hydrated radius and ionic potential of M^{n+}.

Atomic number	Metal ion	Hydrated radius (nm)	Ionic potential (nm^{-1})[a, b]	Reference
3	Li^{+}	0.382	2.618	[19]
11	Na^{+}	0.358	2.793	[19]
47	Ag^{+}	0.341	2.933	[19]
19	K^{+}	0.331	3.021	[19]
25	Mn^{2+}	0.438	4.566	[20]
30	Zn^{2+}	0.430	4.651	[19]
12	Mg^{2+}	0.428	4.673	[19]
26	Fe^{2+}	0.428	4.673	[20]
48	Cd^{2+}	0.426	4.695	[19]
27	Co^{2+}	0.423	4.728	[20]
29	Cu^{2+}	0.419	4.773	[20]
20	Ca^{2+}	0.412	4.854	[19]
28	Ni^{2+}	0.404	4.950	[20]
56	Ba^{2+}	0.404	4.950	[20]
13	Al^{3+}	0.480	6.250	[19]
24	Cr^{3+}	0.461	6.508	[20]
26	Fe^{3+}	0.457	6.565	[20]

[a] Calculated by $\Phi = Z/r$.

[b] Calculated from the hydrated radius instead of ionic radius.

Table S4. Selected the redox couple of metallic element.

Atomic number	Redox couple	$E^{\ominus}$ (V)	Atomic number	Redox couple	$E^{\ominus}$ (V)
20	Ca^{2+}/Ca	−2.84	48	Cd^{2+}/Cd	−0.40
13	Al^{3+}/Al	−1.68	27	Co^{2+}/Co	−0.28
12	Mg^{2+}/Mg	−2.36	28	Ni^{2+}/Ni	−0.26
22	Ti^{2+}/Ti	−1.63	50	Sn^{2+}/Sn	−0.14
3	Li^{+}/Li	−3.04	82	Pb^{2+}/Pb	−0.13
19	K^{+}/K	−2.93	26	Fe^{3+}/Fe	−0.04
11	Na^{+}/Na	−2.71	50	Sn^{4+}/Sn^{2+}	0.15
25	Mn^{2+}/Mn	−1.18	29	Cu^{+}/Cu	0.52
23	V^{2+}/V	−1.13	29	Cu^{2+}/Cu	0.34
24	Cr^{3+}/Cr	−0.74	47	Ag^{+}/Ag	0.80
30	Zn^{2+}/Zn	−0.76	78	Pt^{2+}/Pt	1.19
26	Fe^{2+}/Fe	−0.44	79	Au^{3+}/Au	1.52

Table S5. Antiviral rate of metal elements with different valence states.

Valence	Antiviral rate ($mM^{-1}·h^{-1}$)				Mean
	Example A	Example B	Example C	Example D	
Fe	2.311 [21]	3.807 [22]	4.552 [23]		3.557
Fe^{2+}	0.090 [12]	0.605 [6]			0.347
Fe^{3+}	0.006 [18]	0.041 [18]			0.023
Cu^{+}	0.314 [7]	1.500 [8]	6.190 [9]		2.668
Cu^{2+}	0.029 [5]	1.100 [6]	5.083 [17]		2.070
Ag	1.421 [24]	3.613 [25]	4.201 [26]		3.079
Ag^{+}	0.101 [5]	0.750 [5]	2.080 [6]		0.977
Au	0.576 [27]	5.923 [28]	7.798 [29]	9.319 [29]	5.904
Au^{3+}	0				0

References

1. Shokeen, K.; Srivathsan, A.; Kumar, S. Lithium chloride functions as Newcastle disease virus-induced ER-stress modulator and confers anti-viral effect. *Virus Research* **2021**, *292*, 198223, https://doi.org/10.1016/j.virusres.2020.198223.
2. Li, H.-j.; Gao, D.-s.; Li, Y.-t.; Wang, Y.-s.; Liu, H.-y.; Zhao, J. Antiviral effect of lithium chloride on porcine epidemic diarrhea virus in vitro. *Research in Veterinary Science* **2018**, *118*, 288-294, https://doi.org/10.1016/j.rvsc.2018.03.002.
3. Ren, X.; Meng, F.; Yin, J.; Li, G.; Li, X.; Wang, C.; Herrler, G. Action mechanisms of lithium chloride on cell infection by transmissible gastroenteritis coronavirus. *PloS one* **2011**, *6*, e18669, doi:10.1371/journal.pone.0018669.
4. Sui, X.; Yin, J.; Ren, X. Antiviral effect of diammonium glycyrrhizinate and lithium chloride on cell infection by pseudorabies herpesvirus. *Antiviral Research* **2010**, *85*, 346-353, https://doi.org/10.1016/j.antiviral.2009.10.014.
5. Minoshima, M.; Lu, Y.; Kimura, T.; Nakano, R.; Ishiguro, H.; Kubota, Y.; Hashimoto, K.; Sunada, K. Comparison of the antiviral effect of solid-state copper and silver compounds. *Journal of Hazardous Materials* **2016**, *312*, 1-7, https://doi.org/10.1016/j.jhazmat.2016.03.023.
6. Molan, K.; Rahmani, R.; Krklec, D.; Brojan, M.; Stopar, D. Phi 6 Bacteriophage Inactivation by Metal Salts, Metal Powders, and Metal Surfaces. *Viruses* **2022**, *14*, doi:10.3390/v14020204.
7. Sunada, K.; Minoshima, M.; Hashimoto, K. Highly efficient antiviral and antibacterial activities of solid-state cuprous compounds. *Journal of Hazardous Materials* **2012**, *235-236*, 265-270, https://doi.org/10.1016/j.jhazmat.2012.07.052.
8. Takeda, Y.; Jamsransuren, D.; Nagao, T.; Fukui, Y.; Matsuda, S.; Ogawa, H.; Johnson Karyn, N. Application of Copper Iodide Nanoparticle-Doped Film and Fabric To Inactivate SARS-CoV-2 via the Virucidal Activity of Cuprous Ions (Cu+). *Applied and Environmental Microbiology 87*, e01824-01821, doi:10.1128/AEM.01824-21.
9. Fujimori, Y.; Sato, T.; Hayata, T.; Nagao, T.; Nakayama, M.; Nakayama, T.; Sugamata, R.; Suzuki, K. Novel Antiviral Characteristics of Nanosized Copper(I) Iodide Particles Showing Inactivation Activity against 2009 Pandemic H1N1 Influenza Virus. *Applied and Environmental Microbiology* **2012**, *78*, 951-955, doi:10.1128/AEM.06284-11.
10. Lukasik, J.; Scott Troy, M.; Andryshak, D.; Farrah Samuel, R. Influence of Salts on Virus Adsorption to Microporous Filters. *Applied and Environmental Microbiology* **2000**, *66*, 2914-2920, doi:10.1128/AEM.66.7.2914-2920.2000.
11. Wei, Z.; Burwinkel, M.; Palissa, C.; Ephraim, E.; Schmidt, M.F.G. Antiviral activity of zinc salts against transmissible gastroenteritis virus in vitro. *Veterinary Microbiology* **2012**, *160*, 468-472, https://doi.org/10.1016/j.vetmic.2012.06.019.
12. Tong, T.; Deng, S.; Zhang, X.; Fang, L.; Liang, J.; Xiao, S. Inhibitory effect and mechanism of gelatin stabilized ferrous sulfide nanoparticles on porcine reproductive and respiratory syndrome virus. *Journal of nanobiotechnology* **2022**, *20*, 70, doi:10.1186/s12951-022-01281-4.
13. Sengupta, D.; Timilsina, U.; Mazumder, Z.H.; Mukherjee, A.; Ghimire, D.; Markandey, M.; Upadhyaya, K.; Sharma, D.; Mishra, N.; Jha, T.; et al. Dual activity of amphiphilic Zn(II) nitroporphyrin derivatives as HIV-1 entry inhibitors and in cancer photodynamic therapy. *European Journal of Medicinal Chemistry* **2019**, *174*, 66-75, https://doi.org/10.1016/j.ejmech.2019.04.051.
14. Kaushik, N.; Subramani, C.; Anang, S.; Muthumohan, R.; Shalimar, n.; Nayak, B.; Ranjith-

Kumar, C.T.; Surjit, M.; Ou, J.H.J. Zinc Salts Block Hepatitis E Virus Replication by Inhibiting the Activity of Viral RNA-Dependent RNA Polymerase. *Journal of Virology 91*, e00754-00717, doi:10.1128/JVI.00754-17.

15. Horie, M.; Ogawa, H.; Yoshida, Y.; Yamada, K.; Hara, A.; Ozawa, K.; Matsuda, S.; Mizota, C.; Tani, M.; Yamamoto, Y.; et al. Inactivation and morphological changes of avian influenza virus by copper ions. *Archives of Virology* **2008**, *153*, 1467, doi:10.1007/s00705-008-0154-2.
16. Lin, Y.-C.; Chang, C.-H. In vitro inhibition of enterovirus 71 infection with a nickel ion/chitosan microcomposite. *Virus Research* **2014**, *190*, 17-24, https://doi.org/10.1016/j.virusres.2014.06.012.
17. Soliman, M.Y.M.; Medema, G.; Bonilla, B.E.; Brouns, S.J.J.; van Halem, D. Inactivation of RNA and DNA viruses in water by copper and silver ions and their synergistic effect. *Water Research X* **2020**, *9*, 100077, https://doi.org/10.1016/j.wroa.2020.100077.
18. Terpiłowska, S.; Siwicki, A.K. Chromium(III) and iron(III) inhibits replication of DNA and RNA viruses. *BioMetals* **2017**, *30*, 565-574, doi:10.1007/s10534-017-0027-9.
19. Tansel, B. Significance of thermodynamic and physical characteristics on permeation of ions during membrane separation: Hydrated radius, hydration free energy and viscous effects. *Separation and Purification Technology* **2012**, *86*, 119-126, https://doi.org/10.1016/j.seppur.2011.10.033.
20. Nightingale, E.R. Phenomenological Theory of Ion Solvation. Effective Radii of Hydrated Ions. *The Journal of Physical Chemistry* **1959**, *63*, 1381-1387, doi:10.1021/j150579a011.
21. Cheng, R.; Zhang, Y.; Zhang, T.; Hou, F.; Cao, X.; Shi, L.; Jiang, P.; Zheng, X.; Wang, J. The inactivation of bacteriophages MS2 and PhiX174 by nanoscale zero-valent iron: Resistance difference and mechanisms. *Frontiers of Environmental Science & Engineering* **2022**, *16*, 108, doi:10.1007/s11783-022-1529-4.
22. Cheng, R.; Li, G.; Cheng, C.; Liu, P.; Shi, L.; Ma, Z.; Zheng, X. Removal of bacteriophage f2 in water by nanoscale zero-valent iron and parameters optimization using response surface methodology. *Chemical Engineering Journal* **2014**, *252*, 150-158, https://doi.org/10.1016/j.cej.2014.05.003.
23. Yuan, D.; Zhai, L.; Zhang, X.; Cui, Y.; Wang, X.; Zhao, Y.; Xu, H.; He, L.; Yan, C.; Cheng, R.; et al. Study on the characteristics and mechanism of bacteriophage MS2 inactivated by bacterial cellulose supported nanoscale zero-valent iron. *Journal of Cleaner Production* **2020**, *270*, 122527, https://doi.org/10.1016/j.jclepro.2020.122527.
24. Morris, D.; Ansar, M.; Speshock, J.; Ivanciuc, T.; Qu, Y.; Casola, A.; Garofalo, R.P. Antiviral and Immunomodulatory Activity of Silver Nanoparticles in Experimental RSV Infection. *Viruses* **2019**, *11*, doi:10.3390/v11080732.
25. He, Q.; Lu, J.; Liu, N.; Lu, W.; Li, Y.; Shang, C.; Li, X.; Hu, L.; Jiang, G. Antiviral Properties of Silver Nanoparticles against SARS-CoV-2: Effects of Surface Coating and Particle Size. *Nanomaterials* **2022**, *12*, doi:10.3390/nano12060990.
26. Lv, X.; Wang, P.; Bai, R.; Cong, Y.; Suo, S.; Ren, X.; Chen, C. Inhibitory effect of silver nanomaterials on transmissible virus-induced host cell infections. *Biomaterials* **2014**, *35*, 4195-4203, https://doi.org/10.1016/j.biomaterials.2014.01.054.
27. Kim, J.; Yeom, M.; Lee, T.; Kim, H.O.; Na, W.; Kang, A.; Lim, J.W.; Park, G.; Park, C.; Song, D.; et al. Porous gold nanoparticles for attenuating infectivity of influenza A virus. *Journal of nanobiotechnology* **2020**, *18*, 54, doi:10.1186/s12951-020-00611-8.
28. Meléndez-Villanueva, M.A.; Morán-Santibañez, K.; Martínez-Sanmiguel, J.J.; Rangel-

López, R.; Garza-Navarro, M.A.; Rodríguez-Padilla, C.; Zarate-Triviño, D.G.; Trejo-Ávila, L.M. Virucidal Activity of Gold Nanoparticles Synthesized by Green Chemistry Using Garlic Extract. *Viruses* **2019**, *11*, doi:10.3390/v11121111.

29. Halder, A.; Das, S.; Ojha, D.; Chattopadhyay, D.; Mukherjee, A. Highly monodispersed gold nanoparticles synthesis and inhibition of herpes simplex virus infections. *Materials Science and Engineering: C* **2018**, *89*, 413-421, https://doi.org/10.1016/j.msec.2018.04.005.
